# Glucose 6P Binds and Activates HlyIIR to Repress *Bacillus cereus* Haemolysin *hlyII* Gene Expression

**DOI:** 10.1371/journal.pone.0055085

**Published:** 2013-02-06

**Authors:** Elisabeth Guillemet, Seav-Ly Tran, Céline Cadot, Didier Rognan, Didier Lereclus, Nalini Ramarao

**Affiliations:** 1 INRA, Unité MICALIS UMR-1319, La Minière, Guyancourt, France; 2 CNRS, UMR 7200, Laboratory of Therapeutic Innovation, Illkirch, France; University Medical Center Utrecht, The Netherlands

## Abstract

*Bacillus cereus* is a Gram-positive spore-forming bacterium causing food poisoning and serious opportunistic infections. These infections are characterized by bacterial accumulation despite the recruitment of phagocytic cells. We have previously shown that *B. cereus* Haemolysin II (HlyII) induces macrophage cell death by apoptosis. In this work, we investigated the regulation of the *hlyII* gene. We show that HlyIIR, the negative regulator of *hlyII* expression in *B. cereus*, is especially active during the early bacterial growth phase. We demonstrate that glucose 6P directly binds to HlyIIR and enhances its activity at a post-transcriptional level. Glucose 6P activates HlyIIR, increasing its capacity to bind to its DNA-box located upstream of the *hlyII* gene, inhibiting its expression. Thus, *hlyII* expression is modulated by the availability of glucose. As HlyII induces haemocyte and macrophage death, two cell types that play a role in the sequestration of nutrients upon infection, HlyII may induce host cell death to allow the bacteria to gain access to carbon sources that are essential components for bacterial growth.

## Introduction

The *Bacillus cereus* group is composed of highly related pathogenic species, including *B. thuringiensis*, an insect pathogen, *B. anthracis*, the etiological agent of anthrax and *B. cereus. B. cereus* is an emerging human food-borne pathogen classified as the 3^rd^ most important cause of collective food-borne infections in Europe, after *Salmonella* and *Staphylococcus* [1]. *B. cereus* infection generally causes mild disease characterised by gastroenteritis, but bloody diarrhea and emetic poisoning leading to some fatal cases have been reported [2]. *B. cereus* is also associated with severe local and systemic human infections, posing a public health problem [3]. Infections such as endophthalmitis, pneumonia and meningitis, particularly in neonates in which they may cause death of the infant within days, have been attributed to *B. cereus* [4], [5], [6], [7].

In the early stationary phase, *B. cereus* produces several extracellular compounds (degradative enzymes, cytotoxic factors and cell surface proteins) that might contribute to virulence [8], [9], [10], [11], [12]. However, the mechanisms leading to the various pathologies of *B. cereus* are not completely elucidated. The non-gastrointestinal infections are characterized by bacteremia despite the accumulation of inflammatory cells at the site of infection [13]. This implies that the bacteria have developed means to resist the activity of inflammatory cells and thus the host immune system. Our previous work has consistently shown that *B. cereus* is able to circumvent the host immune response. *B. cereus* spores survive, germinate and multiply in contact with macrophages [14], eventually leading to the production of toxins [15]. Among these toxins, the haemolysin HlyII has been shown to be responsible for macrophage death [15], [16]. HlyII is a member of the oligomeric ß-barrel pore-forming toxins, which include the α-toxin of *Staphylococcus aureus*, the ß-toxin of *Clostridium perfringens* and the *B. cereus* Cytotoxin K (CytK) [17], [18], [19]. HlyII is a secreted protein that induces pore formation in the membrane of various eukaryotic cells [20], [21], [22]. HlyII has haemolytic properties [20], [23] and induces apoptosis of host monocytes and macrophages *in vivo* [15]. When expressed in *B. subtilis,* HlyII induces haemolysis and virulence in a crustacean infection model [24]. We have also demonstrated the importance of HlyII for *B. cereus* virulence in insects and mice [15]. The importance of HlyII has been strengthened by the fact that the *hlyII* gene is present in several clinical isolates of *B. cereus* [25].

A precise knowledge of the conditions governing the expression of a gene improves our understanding of its role in the functioning of the bacterial cell, but also during pathogenesis. The regulation of *hlyII* expression has so far been exclusively studied in the heterologous systems *E. coli* and *B. subtilis*. In those systems, it has been shown that *hlyII* expression is negatively regulated by the transcriptional regulator HlyIIR [26]. The *hlyII* and *hlyIIR* genes are situated in the same chromosomal locus (Bc 3523 and Bc 3522 in the ATCC 14579 strain, respectively) but are not organized in an operon. *In vitro* analyses have shown that recombinant HlyIIR regulates *hlyII* expression by specific binding, as two dimers, to a 44 perfect inverted DNA repeat (22 bp x 2) centred 48 bp upstream of the *hlyII* transcription initiation point [27].

In this study, we investigated the regulatory process of *hlyII* expression and of its repressor *hlyIIR*. We highlight the major role of glucose 6P as a post-transcriptional activator of HlyIIR, and show that glucose 6P binds to and activates HlyIIR to inhibit *hlyII* expression.

## Materials and Methods

### Bacterial Strains

The *B. thuringiensis* 407 Cry^_^ strain was used as a model for *B. cereus*. This sequenced strain was originally described as a *B. thuringiensis* strain, but cured of its plasmid it is acrystalliferous and shows high phylogenic similarity with the *B. cereus* reference strain ATCC 14579 [28]. The Bt407 strain was used as the wild type background and transformed as previously described [29].

### Plasmid and Mutant Strain Constructions

The *hlyIIR* gene was disrupted as follows. *Hind*III-*Xba*I (1024 bp) and *Sph*I-*Bam*HI (964 bp) DNA fragments corresponding to upstream and downstream regions of the *hlyIIR* gene were generated from the Bt407 chromosome by PCR using the primer pairs:

hlyIIR-1 (5′-CCCAAGCTTGTACCAGCTTTAACAGGCTATGG -3′),

hlyIIR-2 (5′- GCTCTAGACGTCTGCTCTCGAGATTTCCCC-3′), and

hlyIIR-3 (5′-ACATGCATGCGGATGCCAGAAGACTTAGTGG -3′),

hlyIIR-4 (5′-CGGGATCCCAGGCTCTAAGTTGGATAAGGGG -3′).

A Tet^R^ cassette carrying a *tet* gene was purified from pHTS1 [30] as a 1.6 Kb (*Xba*I and *Sph*I) fragment. The amplified DNA fragments were digested with the appropriate enzymes and inserted between the *Hind*III and *Bam*HI sites of pRN5101, with the selection marker cassette cloned in between the up- and downstream chromosomal fragments of the *hlyIIR* gene. The resulting plasmid was introduced into Bt407 by electroporation [31] and the *hlyIIR* gene was deleted by a double crossover event as previously described [32]. Chromosomal allele exchange was confirmed by PCR with oligonucleotide primers located upstream from hlyIIR-1: hlyIIR-5 (5′-GGGCCAAATGCCGATGCAAGTATCACAGGTAGC-3′) and downstream from hlyIIR-4: hlyIIR-6 (5′-CCAAGCGTTCAGCAAGTTGGTCTCATGATGTGG-3′), and in the Tet^R^ cassette (5′-CGGGTCGGTAATTGGGTTTG-3′), (5′-GCAGCTGCACCAGCCCCTTG-3′). The mutant strain was designated Bt407Δ*hlyIIR.*

Transcriptional *hlyII-lacZ* and *hlyIIR-lacZ* fusions were constructed using DNA fragments corresponding to the promoter regions of *hlyII* and *hlyIIR*, generated by PCR using the primer pair PhlyII-1 (5′- AAAACTGCAGCCAGCTGTGTTGACAGAACTGGC-3′) and PhlyII-2 (5′-GCTCTAGACGGACGCTACCGCAACGCATTTAGC -3′), and hlyIIR-1 and hlyIIR-2, respectively. The PCR fragments were digested with the appropriate enzymes and inserted between the *Xba*I and *Pst*I sites of pHT304-18Z [33]. The recombinant plasmids, designated pHT-P*hlyII’Z* and pHT-P*hlyIIR’Z* were introduced into Bt407 and into the mutant strain Bt407Δ*hlyIIR* by electroporation. Transformants were named Bt407 [pHT-P*hlyII’Z*], Bt407 [pHT-P*hlyIIR’Z*] and Bt407Δ*hlyIIR* [pHT-P*hlyII’Z*].

### Purification of the HlyIIR Protein

The plasmid pGEX6P1-GST-HlyIIR was constructed as follows. The *hlyIIR* gene was amplified from the Bt407 chromosome by PCR using the primer pairs hlyIIR-GST-1 (5′-CGGGATCCATGGGGAAATCTCCAGAGCAGACGA-3′) and hlyIIR-GST-2 (5′-TCCCCCGGGCCCGTATGCAAATCGAAGAGCTTAT-3′). The corresponding DNA fragment was inserted between the *BamH*I and *Sma*I sites of plasmid pGEX6P1 (GE Healthcare), and the resulting plasmid was introduced into *E. coli* M15^hello^pREP4] (Qiagen). *E. coli* M15^hello^pREP4] strain harboring the plasmid pGEX6P1-GST-HlyIIR was grown in minimal M9 medium [34] with 3% arabinose as unique carbon source, at 37°C until OD_600_ 0.8 was reached and protein expression was induced by addition of 1 mM IPTG. Growth was continued for 4 h after IPTG induction. Bacteria were then collected by centrifugation at 7700 g for 10 min. Pellets were lyzed using 1% triton X-100 and sonication in PBS. After centrifugation, the tagged HlyIIR contained in the supernatant was added on a Bulk GST Purification module (GE Healthcare). The HlyIIR protein was purified by cleavage of the GST tag using the Precission protease according to the manufacturer’s instructions. Protein concentration was calculated by Bradford staining and purity was assessed on SDS-Page gel.

### Relative Quantification of *hlyII* Gene Expression by RT-qPCR

#### Preparation of the RNA samples

Bt407 and Bt407Δ*hlyIIR* were cultivated aerobically in LB at 37°C with agitation.

1 ml samples were taken every hour between t0−2 h (t−2) and t0+4 h (t4), t0 h (t0) corresponding to the transition state between the exponential and the stationary phases of the bacterial culture. The bacterial cells were collected by a short centrifugation and immediately placed at −80°C. Total RNA of these cell pellets was extracted as described in [25]. Briefly, cells were thawed and lysed in Tri-reagent (Ambion), RNA was purified using the RNeasy kit (Qiagen) and finally treated with DNase (TURBO DNA-free kit; Ambion). Analyses of RNA solutions prepared according to this protocol have shown that they are of a high purity grade (OD_260 nm_/OD_280 nm_ >1,95 and RIN >9) and free of DNA traces (data not shown).

#### RT-qPCR experiments

Target and reference gene mRNA abundance was measured by one-step RT-qPCR with the QuantiFast SYBR green RT-PCR kit, following the manufacturer’s instructions (Qiagen). We used the *ssu* gene, encoding 16S RNA, as endogenous reference, as its expression level in Bt407 was stable between t-2 and t4 (data not shown). RT-qPCR samples contained 1 ng of RNA and 500 nM or 1 µM of each primer in a final volume of 25 µL and were run in a LightCycler 480 (Roche). The following primer pairs were used: F-hlyII-q (5′-CTGGAAAAACCATCAAGTTACTC-3′); R-hlyII-dq (5′-TCACCATTTACAAAGATACC-3′) [25] and LC-16S-F (5′-GGTAGTCCACGCCGTAAACG-3′); LC-16S-R (5′-GACAACCATGCACCACCTG-3′) [35]. The efficiency of both primer pairs in the experiment conditions was above 95% (data not shown). Expression levels of the target gene relative to the endogenous standard were calculated using the basic relative quantification method (Roche) and the LightCycler 480 software. The specificity of each amplification reaction was verified with the melting curve profile: a single peak was obtained, attesting to the amplification of a unique product.

### ß-galactosidase Assays

Strains harboring plasmid transcriptional *lacZ* fusions were grown in LB medium at 37°C under shaking. Samples were taken every hour from t−2 to t4. Determination of ß-galactosidase activity was achieved as previously described [36], [37]. When indicated, 0.3% glucose or arabinose was added to the medium at t−1.

### Molecular Docking

The crystal structure of *B. cereus* HlyIIR was retrieved from the Protein Data Bank (PDB entry 2fx0) and hydrogen atoms added with the Biopolymer module of the SYBYL-X 1.3 package (Tripos Assoc. Inc, St.Louis, USA). The starting three-dimensional structure of glucose-6-Phosphate (glucose 6P) was built from the SYBYL fragment library. Docking of the ligand to the HlyIIR cavity was realized using two independent docking tools (PLANTS, GOLD).

PLANTS [38] was used in its 1.2 version with default settings. The geometric center of the HlyIIR cavity was used to define the binding site including any protein atom within 15 Å of these coordinates. Poses were scored with the ChemPLP function and up to 10 solutions were saved for the ligand.

GOLD v5.1 [39] was used to confirm previous PLANTS results, using the same binding site definition as for PLANTS. Default docking settings were applied to dock the ligand and 10 poses were saved.

### Microcalorimetry

Isothermal titration calorimetry (ITC) measures heat generated or absorbed upon the binding of two molecules [40], and yields in a single experiment the thermodynamic parameters allowing the calculation of a dissociation constant (Kd). Isothermal titration calorimetry experiments were performed at 20°C with an ITC200 isothermal titration calorimeter (Microcal®). The interaction between HlyIIR and glucose 6P was tested. The protein concentration in the microcalorimeter cell was set at 28 µM. glucose 6P was resuspended at 0,9 mM in the protein storage buffer. A total of 20 injections of 2 µl of glucose 6P were made at intervals of 180 s while stirring at 800 rpm. Arabinose at 0.9 mM was used as control. We then tested the interaction between DNA and HlyIIR in the absence and presence of glucose 6P (or arabinose). The DNA probe (220 bp) was generated from the Bt407 chromosome by PCR using the following primer pair: 5′-GGAAATAATGTATCTGAATATAGGCTG-3′ and 5′-CACATGTTTCACTAATCTTCCTCTCAC-3′, and containing the HlyIIR binding region upstream of the *hlyII* gene [27]. The HlyIIR concentration in the microcalorimeter cell was set at 28 µM without or with glucose 6P at 1.7%. A total of 20 additions of 2 µl of DNA at 1,25 µM were made by injection at intervals of 180 s while stirring at 800 rpm.

The data were integrated to generate curves in which the areas under the injection peaks were plotted against the ratio of injected sample to cell content. Analysis of the data was performed using MicroCal Origin provided by the manufacturer according to the one-binding-site model. Changes in the free energy and entropy upon binding were calculated from determined equilibrium parameters using the equation:
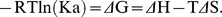
where R is the universal gas constant (1.9872 cal mol-1K-1), T is the temperature in Kelvin degrees, Ka is the association constant, ΔG is the change in Gibbs free energy, ΔH is the change in enthalpy and ΔS is the change in entropy. The binding constant of each interaction is expressed as 1/Ka = Kd (in mol l−1).

## Results

### HlyIIR Negatively Regulates *hlyII* Expression in *B. cereus*

**Figure 1:**
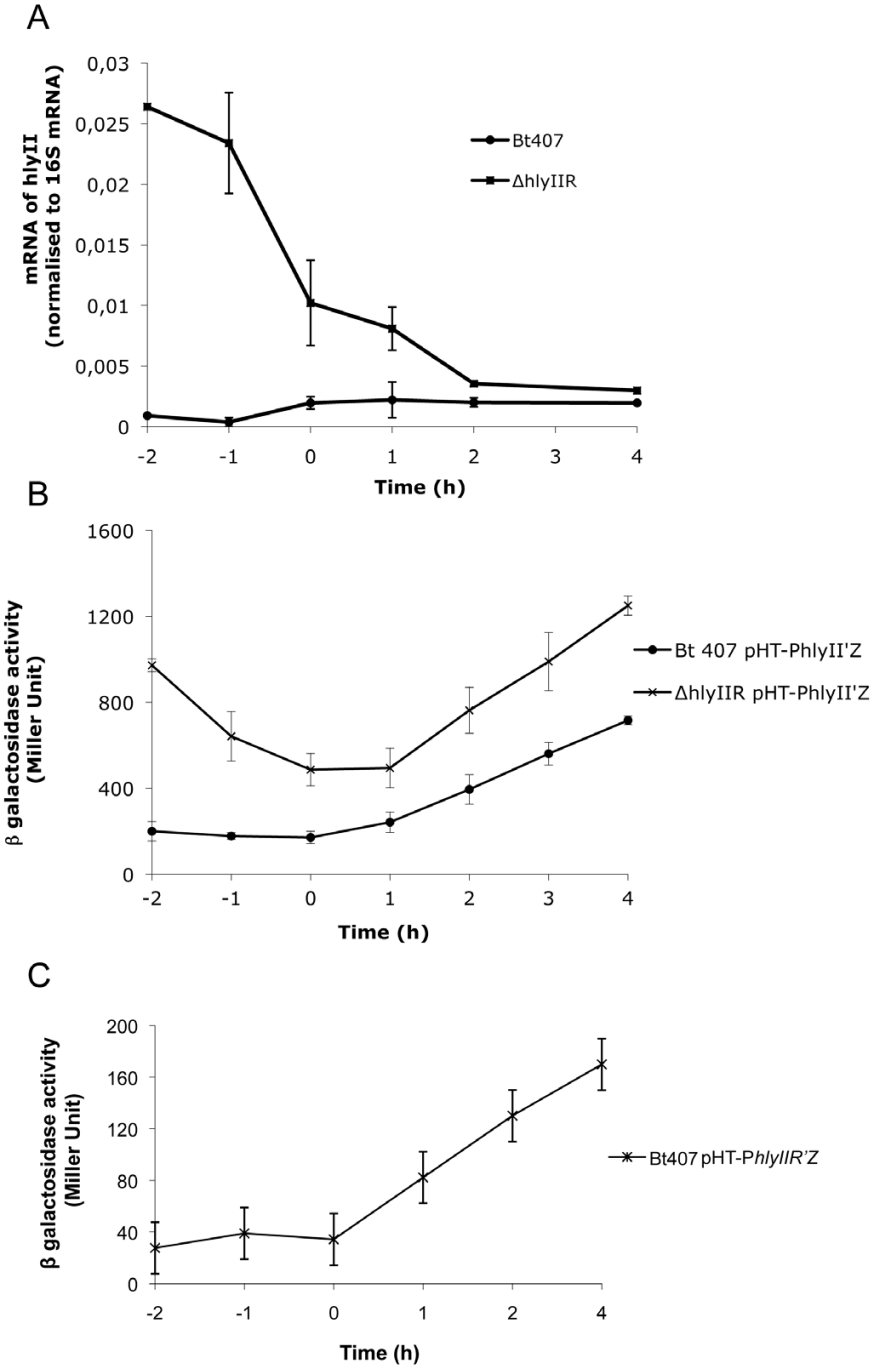
HlyIIR negatively regulates *hlyII* expression in *B. cereus*. (A) Expression levels of the target *hlyII* gene relative to the endogenous standard 16S RNA were measured by RT-qPCR throughout bacterial growth in the wild type Bt407 (circle) and the Bt407Δ*hlyIIR* (square) strains. Data are expressed as the ratio of *hlyII* mRNA normalized to 16S RNA. Values are means of two independent experiments. (B) The specific β-galactosidase activity (Miller unit) of strains Bt407 and Bt407 Δ*hlyIIR* harboring the transcriptional pHT-P*hlyII*’*Z* fusion were measured from bacteria grown in LB medium at 37°C from 2 h before the culture entry into stationary phase (t−2) to 4 h after (t4). Results represent mean values of at least three independent experiments. (C) The specific β-galactosidase activity (Miller unit) of strain Bt407 harboring the pHT-P*hlyIIR*’*Z* fusion was measured from bacteria grown in LB medium at 37°C from 2 h before the culture entered into stationary phase (t−2) to 4 h after (t4). Results represent mean values of at least three independent experiments.

The transcription of *hlyII* is independent of the pleiotropic regulator PlcR [41]. Several studies using heterologous hosts have shown that HlyIIR is a negative regulator of *hlyII* gene transcription. However, no study is so far describing *hlyII* expression regulation by HlyIIR from the *B. cereus* group. We therefore investigated the expression of *hlyII* in a Bt 407 *hlyIIR* deficient mutant. Quantitative RT-PCR assays showed that *hlyII* expression was significantly higher in the Bt407Δ*hlyIIR* mutant than in the wild type (wt) strain (Figure 1A). This difference in expression was most pronounced during exponential phase. The ratio of *hlyII* expression in the Δ*hlyIIR* mutant compared to the wt was 30 fold at t−2, dropped to 5 fold at t0 and stayed around 2 fold after t2. Thus, HlyIIR is a negative regulator of *hlyII* expression in *B. cereus* and is especially active during the early phase of bacterial growth.

The results obtained by qRT PCR were confirmed by ß–galactosidase assays (Figure 1B). The tendency observed was the same, with an increased effect of HlyIIR at the early growth phase (t−2 and t−1), although the ratio measured by ß–galactosidase assay was lower (5 fold) than the one observed by qRT-PCR. However, the ß–galactosidase technique is less precise than qRT-PCR to measure gene expression in the early growth phase due to a small amount of bacteria. After t0, *hlyII* expression was 2 fold higher in the Δ*hlyIIR* mutant than in the wild type strain.

To determine whether the strong effect of HlyIIR on *hlyII* expression during exponential growth phase was due to an increased expression of the regulator itself during this period, we assessed *hlyIIR* expression using a β–galactosidase assay (Figure 1C). *hlyIIR* was expressed during vegetative growth and its expression increased at the onset of stationary phase. These data suggest that HlyIIR-dependent repression of *hlyII* expression during exponential growth phase does not depend on *hlyIIR* gene expression, but likely relies on the activation of the repressor at the protein level.

### Glucose Activates HlyIIR at the Post-transcriptional Level to Inhibit *hlyII* Expression

**Figure 2:**
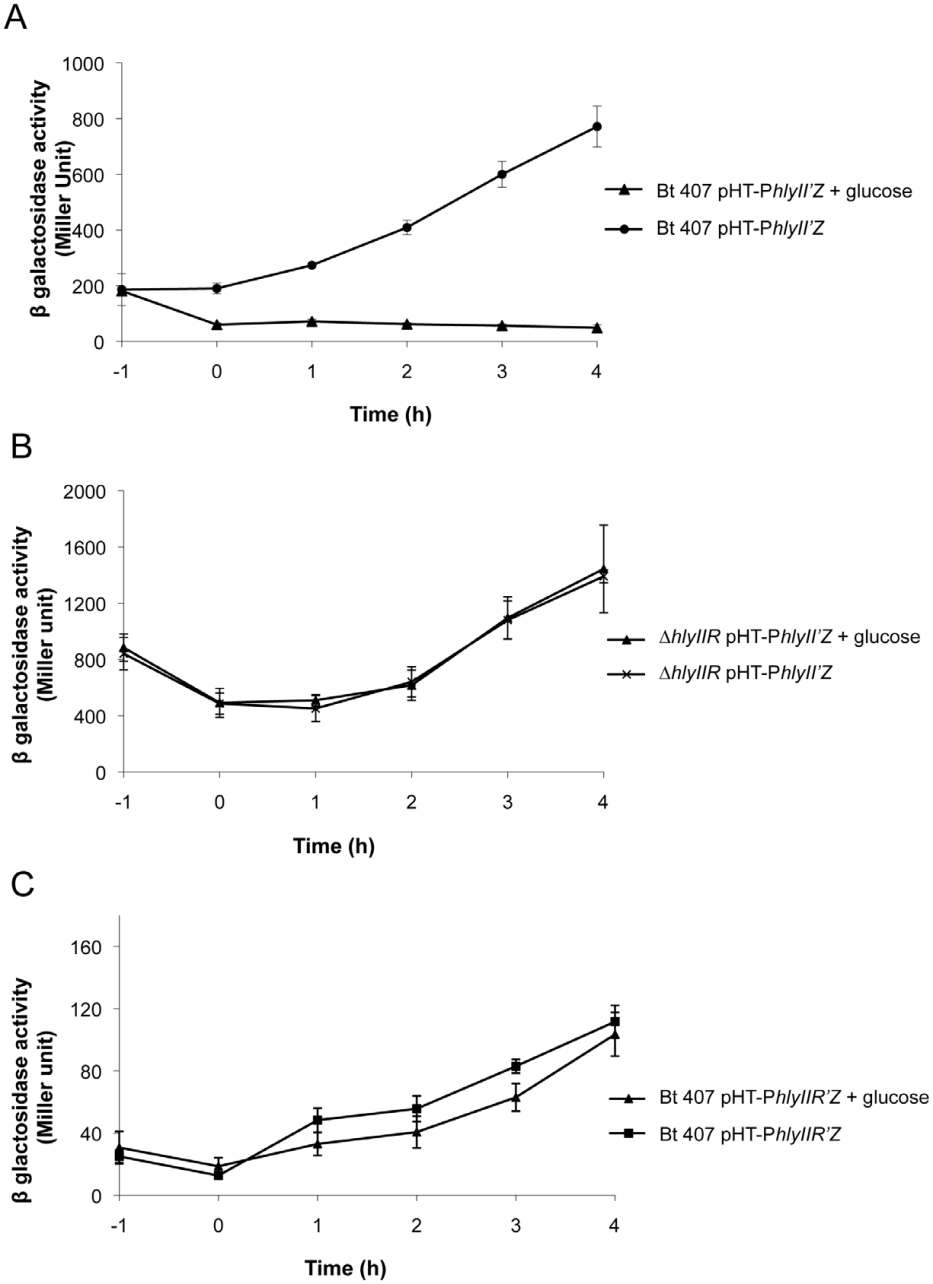
Glucose inhibits *hlyII* expression through activation of the HlyIIR repressor. The bacterial strains Bt407 (A, C) and Bt407*ΔhlyIIR* (B) harboring the transcriptional pHT-P*hlyII’Z* (A, B) or pHT-P*hlyIIR’Z* (C) fusions were grown at 37°C in either LB medium or LB medium supplemented at t−1 with 0.3% glucose. Specific β-galactosidase activity (Miller units) was measured at the indicated time points. Values are expressed as the mean of three independent experiments.

HlyIIR negatively regulates *hlyII* expression, especially during early steps of bacterial growth. We thus investigated whether a co-factor, present during exponential phase and decreasing throughout bacterial growth, could activate HlyIIR. Glucose is a carbon source that is commonly used and consumed by bacteria during bacterial growth. To assess the potential role of glucose on the activation of HlyIIR, we first tested whether glucose had an effect on *hlyII* expression by β–galactosidase assay (Figure 2A). Expression of *hlyII* was completely abolished when glucose was added to the culture, showing that glucose inhibited *hlyII* expression. Not all sugars displayed this activity, as addition of arabinose had no effect on *hlyII* expression (data not shown).

To determine whether glucose inhibitory effect on *hlyII* expression acted through the repressor HlyIIR, expression of *hlyII* was assessed in the presence or absence of glucose in the Δ*hlyIIR* mutant (Figure 2B). The expression of *hlyII* in the Δ*hlyIIR* mutant was similar in the presence and absence of glucose. Thus, in the absence of HlyIIR, glucose had no effect on *hlyII* expression, showing that glucose acted on *hlyII* expression through its repressor HlyIIR.

To assess whether glucose controls *hlyIIR* transcription, *hlyIIR* expression was measured in the presence and absence of glucose (Figure 2C). Addition of glucose to the culture did not significantly alter *hlyIIR* expression. These data show that glucose is not involved in the control of *hlyIIR* expression and strongly suggest that glucose affects *hlyII* expression by activating HlyIIR at a post-transcriptional or a post-translational level.

### Virtual Docking of Glucose 6P in the HlyIIR Ligand Pocket

**Figure 3:**
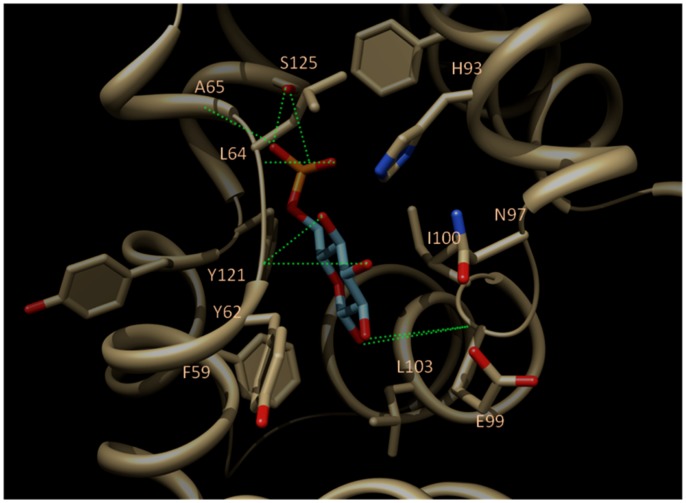
Predicted binding mode common to PLANTS and GOLD docking of glucose-6P (cyan sticks) to the ligand-binding cavity of HlyIIR (yellow ribbons, yellow sticks). Main receptor-interacting residues are labelled at their C-αatoms. Intermolecular hydrogen bonds are displayed as green broken lines. Nitrogen and oxygen atoms are coloured in blue and red, respectively.

The crystal structure of HlyIIR reveals a large internal hydrophobic cavity that could accommodate a ligand with a molecular mass of up to 500 Da, suggesting a co-factor-dependent activation for the inhibition of *hlyII* expression [42]. When glucose is added to the bacterial culture, it is transported inside the bacteria through the complex sugar transporting phosphotransferase system (PTS) [43]. Glucose is then rapidly transformed into glucose 6P, which is thus likely to be the actual effector. To check whether glucose 6P may bind to the above-defined ligand cavity, the compound was docked using two independent state-of-the-art docking algorithms [38], [39]. Interestingly, both programs agreed to unambiguously dock glucose 6P in a single binding mode (Figure 3): an extensive network of 8 hydrogen-bonds to the receptor is found between hydroxyl groups of glucose 6P and main chain carbonyl atoms of HlyIIR (Y62, E99), as well as between the terminal phosphate moiety and the receptor residues (L64, A65, S125). Apolar contacts between the cyclic ring of the ligand and several apolar receptor side chains (F59, Y62, I100, L103) ensure the tight fit of glucose 6P within the binding pocket.

### Direct Binding of Glucose 6P to HlyIIR

**Figure 4:**
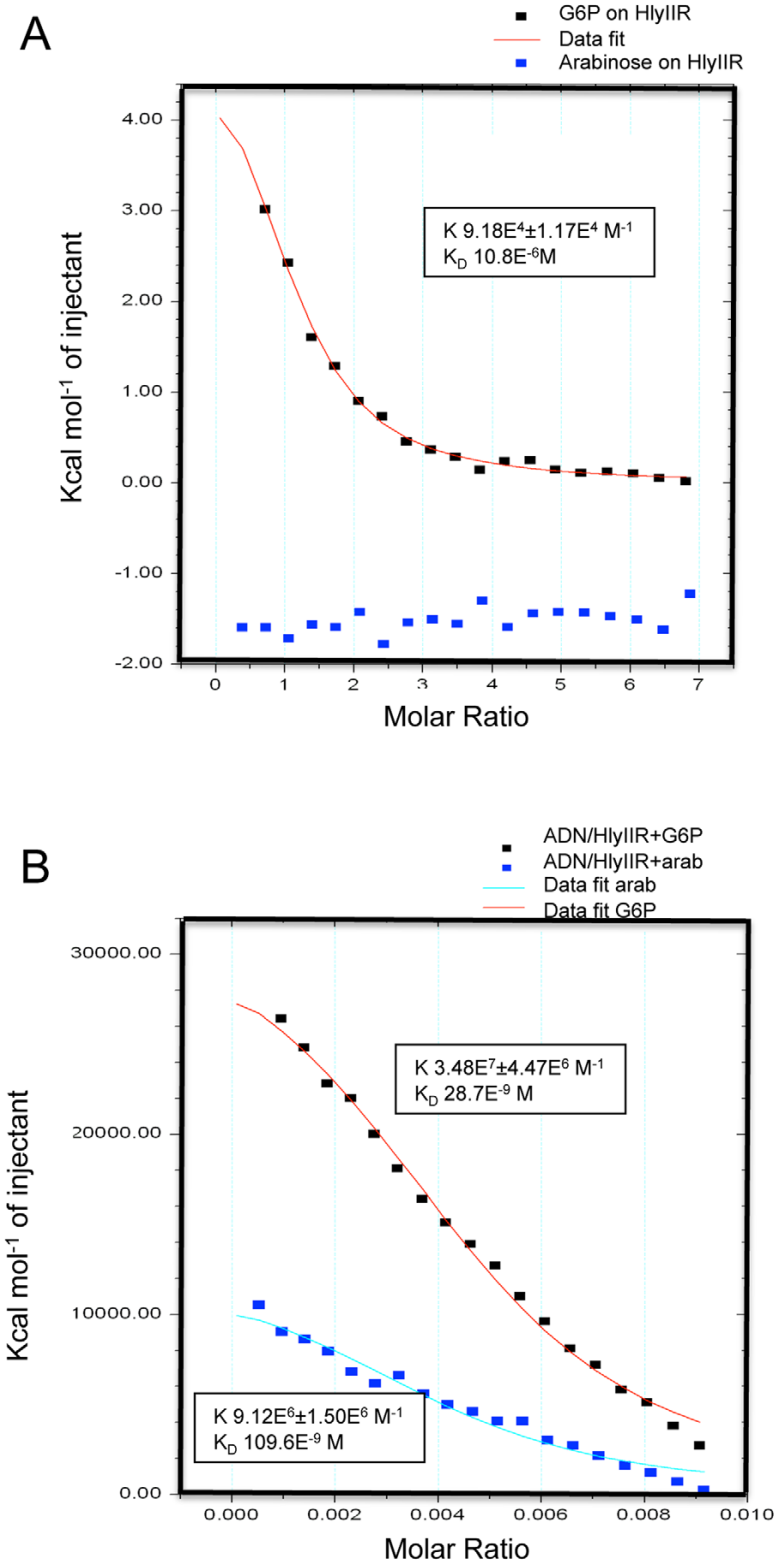
Glucose 6P binds to HlyIIR to enhance its DNA binding capacity. (A) Isothermal titration calorimetry (ITC) assays were performed to measure heat variations generated upon binding between glucose 6P (G6P) and HlyIIR (red line) or arabinose and HlyIIR (blue squares). (B) ITC assays were performed to measure binding between a DNA fragment containing the HlyIIR binding site and HlyIIR supplemented either with glucose 6P (red line) or with arabinose (blue line).

We then used isothermal titration calorimetry (ITC) to confirm and quantitatively characterize the interaction of glucose 6P with HlyIIR (Figure 4A). The HlyIIR protein was produced and purified from a medium lacking glucose or one of its derivatives, and the interaction with glucose 6P was assessed. The analysis of the integrated titration curve showed that the glucose 6P-HlyIIR interaction was characterized by a Kd of about 10.8 µM with an apparent stoichiometry of 1∶1 (N = 1.09±0.00844). These data demonstrate that glucose 6P directly binds to HlyIIR and that one molecule of glucose 6P binds to one monomer of HlyIIR. In sharp contrast, arabinose that we had previously demonstrated having no activation effect on HlyIIR (β-galactosidase assay, not shown), did not either bind HlyIIR in our affinity-binding assay (Figure 4A). This result strongly fosters our hypothesis of glucose specificity within the sugar family of molecules for HlyIIR activation.

### Binding of Glucose 6P to HlyIIR Increases its Affinity to its Target DNA in the *hlyII* Promoter Region

The N-terminal part of HlyIIR presents sequence similarity to regulators of the TetR repressor family which share common features such as a highly conserved helix-turn-helix motif implicated in DNA binding, dependence of cofactors for activity regulation, involvement in the adaptation to a changing environment and acting as homodimers. In view of the 3-D structure of HlyIIR and analogy with its structural homologues TetR, it has been proposed that binding of the appropriate ligand to HlyIIR could change the orientation of the DNA-binding domains leading to a different affinity of the ligand-bound form towards the specific DNA site [42].

We used ITC experiments to characterize whether the DNA binding affinity of HlyIIR is influenced by the presence or absence of glucose 6P binding. Calorimetric titrations were performed and revealed that the HlyIIR- glucose 6P complex binds to the specific DNA target with a Kd of about 28.7 nM (Figure 4B). In the absence of glucose 6P (not shown) or in the presence of arabinose, HlyIIR binds to its target DNA with a Kd of about 110 nM. Thus, in the absence of glucose 6P, HlyIIR was still able to bind to its target DNA but its affinity was increased around 4 times in the presence of glucose 6P.

So far, the HlyIIR-binding ligand was unknown. Thus, to our knowledge, we present the first evidence that HlyIIR trigger its repression activity on *hlyII* expression through its activation by binding glucose 6P.

## Discussion

In this study, we show that Glucose 6P binds and activates HlyIIR, the negative transcriptional regulator of *hlyII* expression in *B. cereus*. Glucose has been shown to be one of the preferred carbon sources during the initial growth phase of *B. subtilis* [44]. At this stage, glucose derivative, Glucose 6P, will activate HlyIIR probably by binding to its ligand cavity. Glucose 6P has been described as an important phosphate donor, leading to activation of various regulators mainly implicated in glycolysis and/or virulence [45], [46]. However, the ability of glucose 6P to bind as a whole into a specific ligand pocket or a regulator implicated in virulence has, to the best of our knowledge, never been described. Thus, we highlight a new mechanism of activation of a transcriptional regulator involved in the regulation of a virulence gene. Other examples have been described of sugars binding to and modulating the activity of repressors implicated in the regulation of genes involved in carbon metabolism, stress response and pathogenesis [47], [48]. For example, in the presence of arabinose, AraC activates the transcription from the promoters of the catabolic operons of various genes, linking the mechanism of glycolysis with virulence [49]. For *B. anthracis*, glucose is a signalling molecule linking environmental sensing to virulence factor production by the way of the important transcription regulator of Gram-positive bacteria, CcpA [50]. CcpA-mediated glucose sensing is also involved in the expression of the *nhe* and *hbl* operons, two major enterotoxins of *B. cereus*, but not in *hlyII* expression [51]. The discovery and characterization of transcription factors mediating the glucose response demonstrate that glucose, like fatty acids and other key nutrients, can directly control gene expression in response to glycaemic variation [52].

**Figure 5:**
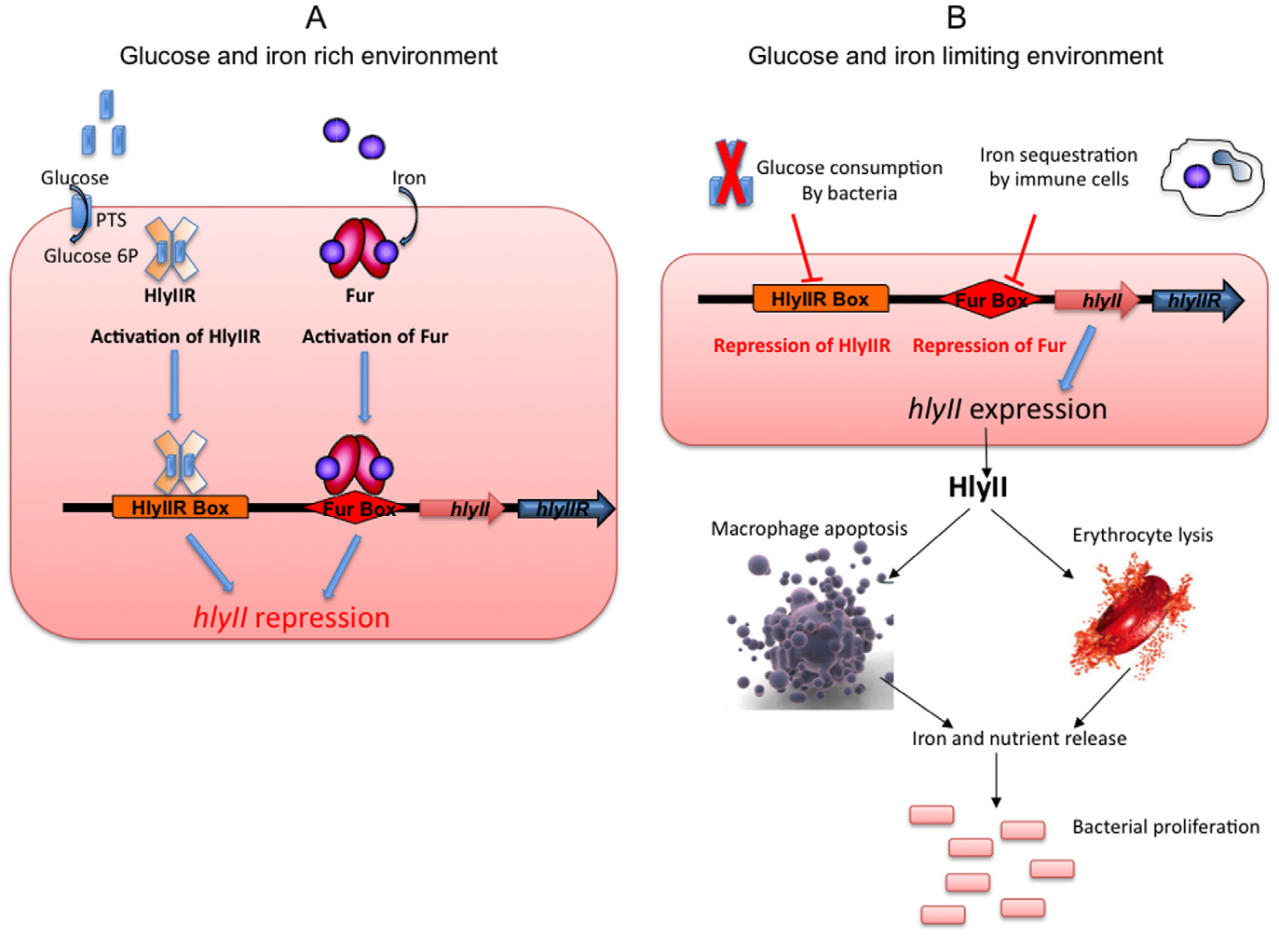
Model of the role and expression of *hlyII* during infection. A) As long as iron and glucose are abundant in the bacterial environment, the bacteria will be able to use these resources for growth. Glucose will enter the bacteria as glucose 6P (blue rectangles) and will bind HlyIIR (orange plain cross). Iron (purple circles) will bind Fur (red ovals). These bindings will promote HlyIIR and Fur repressor activities, leading to HlyIIR- and Fur-based transcriptional repression on *hlyII* gene expression. B) During host infection, bacteria find themselves in an environment, which is low in glucose and iron. Levels of these nutrients are further lowered during bacterial proliferation. The decrease in the concentration of glucose during bacterial proliferation will lead to an inhibition of HlyIIR activity, thus allowing *hlyII* expression. The decrease in the concentration of iron, partially due to its sequestration by immune cells, will lead to an inhibition of Fur activity, thus allowing *hlyII* expression. Thus, when glucose and iron are getting scarce, *hlyII* expression is activated. HlyII will then be released in the environment and induce macrophage and erythrocyte lysis. The dead cells will release their intracellular content, allowing access to metabolites that are essential for bacterial growth.

Taken together, we have shown that HlyIIR regulates *hlyII* gene expression through its post-transcriptional activation by glucose 6P. It has also been shown that *hlyII* expression depends on iron via activation of the global regulator Fur [53]. Indeed, a Fur box was found in the promoter region of the *hlyII* gene [54], and recent data show that Fur binding to this Fur box competes with RNA polymerase binding to the *hlyII* promoter, thus interfering with *hlyII* expression *in vitro* [53]. Sugar and iron are crucial compounds for bacterial multiplication and thus for their capacity to colonize their hosts. It has been reported that a common host defense mechanism relies on iron sequestration by immune cells [55], [56]. An increase in production of iron-binding proteins is observed following infection in order to limit bacterial growth [55]. To counteract this phenomenon, bacteria induce the expression of genes that are negatively iron-regulated. Moreover, in some cases, pathogenic bacteria may acquire iron and sugars/carbohydrates from the host by killing host cells. Thus, the regulation of virulence determinants implicated in host cell death might be regulated by nutrient availability. As a model we propose that when glucose is consumed by the bacteria and iron gets sequestered by phagocytic cells as a natural host defense [55], [56], the HlyIIR and Fur repressors become inactivated and *hlyII* expression starts (Figure 5). HlyII is then produced by the bacteria, secreted and induces haemocyte and macrophage death [15]. The cell content is then released into the environment, providing the bacteria with access to nutrients, allowing bacterial growth and promoting a new cycle of *hlyII* gene inhibition/expression (Figure 5).

Taken together, we have identified several mechanisms triggering *hlyII* gene expression regulation, which are consistent with the role of the HlyII protein during the *B. cereus* infection process. This tight regulation probably limits HlyII production. It is however not clear why *hlyII* gene expression is so tightly regulated by one global (Fur) and one specific (HlyIIR) regulator. Several hypotheses seem possible: i) the low production of HlyII may be sufficient to promote nutrient access and virulence. We have indeed shown that very low doses of protein are able to induce macrophage lysis and mice mortality [15]; ii) HlyII production should remain low to avoid rapid degradation of macrophages and/or host death in order to allow bacterial multiplication and proliferation inside its host.

Additionally, it has recently been shown that HlyII accumulates in high oxidation-reduction potential (ORP) conditions in contrast to low ORP conditions, suggesting a redox-dependent regulation of HlyII secretion [57]. As low ORP anoxic conditions mimics the intestinal environment, HlyII may not be a virulence factor involved in gastrointestinal disease. We have consistently shown that HlyII is involved specifically in immune cell death by apoptosis [15], [16] and that it is highly produced by strains of human clinical origin [25], strongly suggesting a role during opportunistic infections.

Taken together, our data provide new insights into the regulatory pathways of *hlyII*, which plays an important role during the *B. cereus* virulence process. We demonstrate a new role of glucose 6P as a direct activator of a transcriptional regulator. As iron and carbon sources are essential and universal bacterial growth components, we believe that our data provide an important contribution to the understanding of bacterial infection strategies.
